# Supplementary figures and images for: Establishment of a CPER reverse genetics system for Powassan virus defines attenuating NS1 glycosylation sites and an infectious NS1-GFP11 reporter virus
Source: mBio. 2023 Jul 25;14(4):e01388-23. doi: 10.1128/mbio.01388-23 (PMC10470542; doi:10.1128/mbio.01388-23)

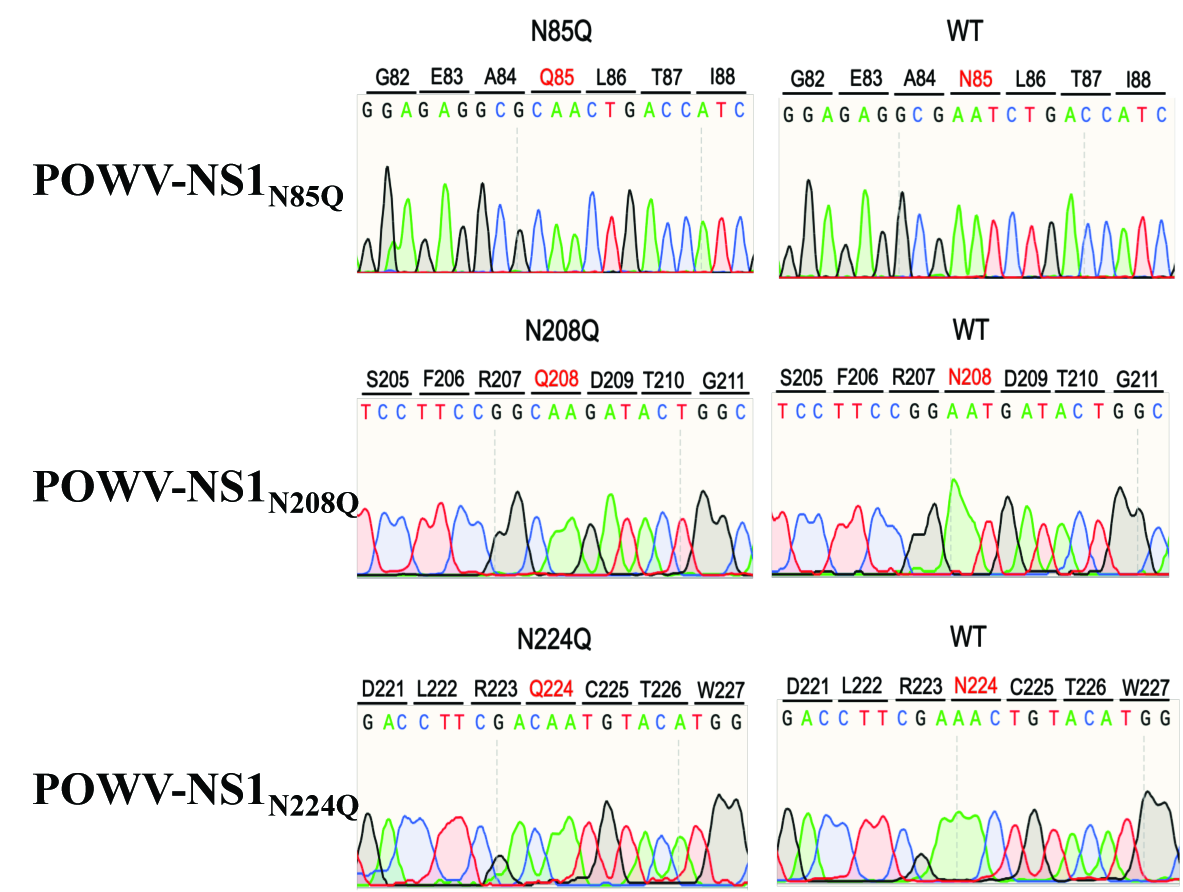

Supplement: Fig. S1 — Sequencing chromatograms. [file mbio.01388-23-s0001.tif]

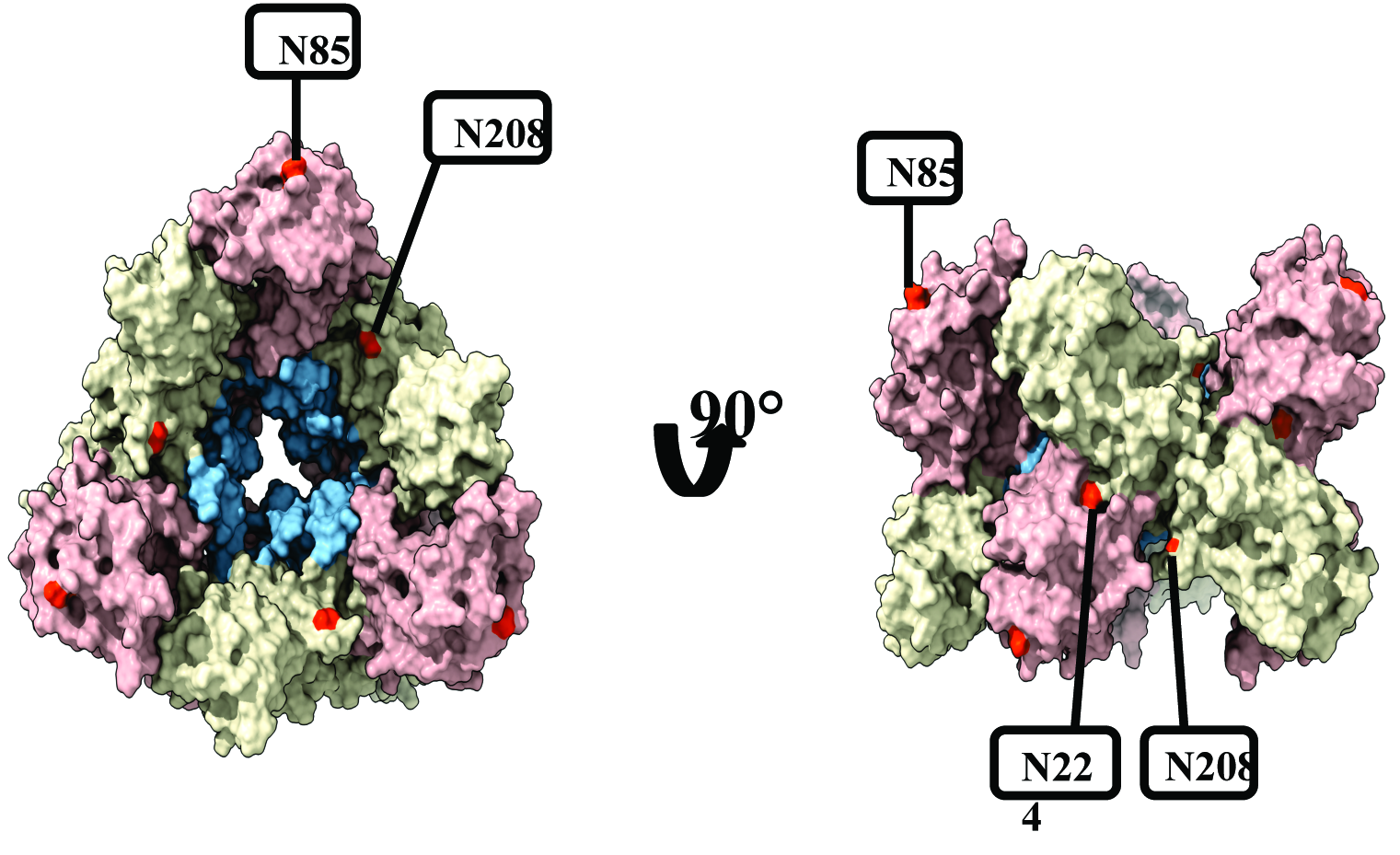

Supplement: Fig. S2 — POWV NS2 hexamer model. [file mbio.01388-23-s0002.tif]

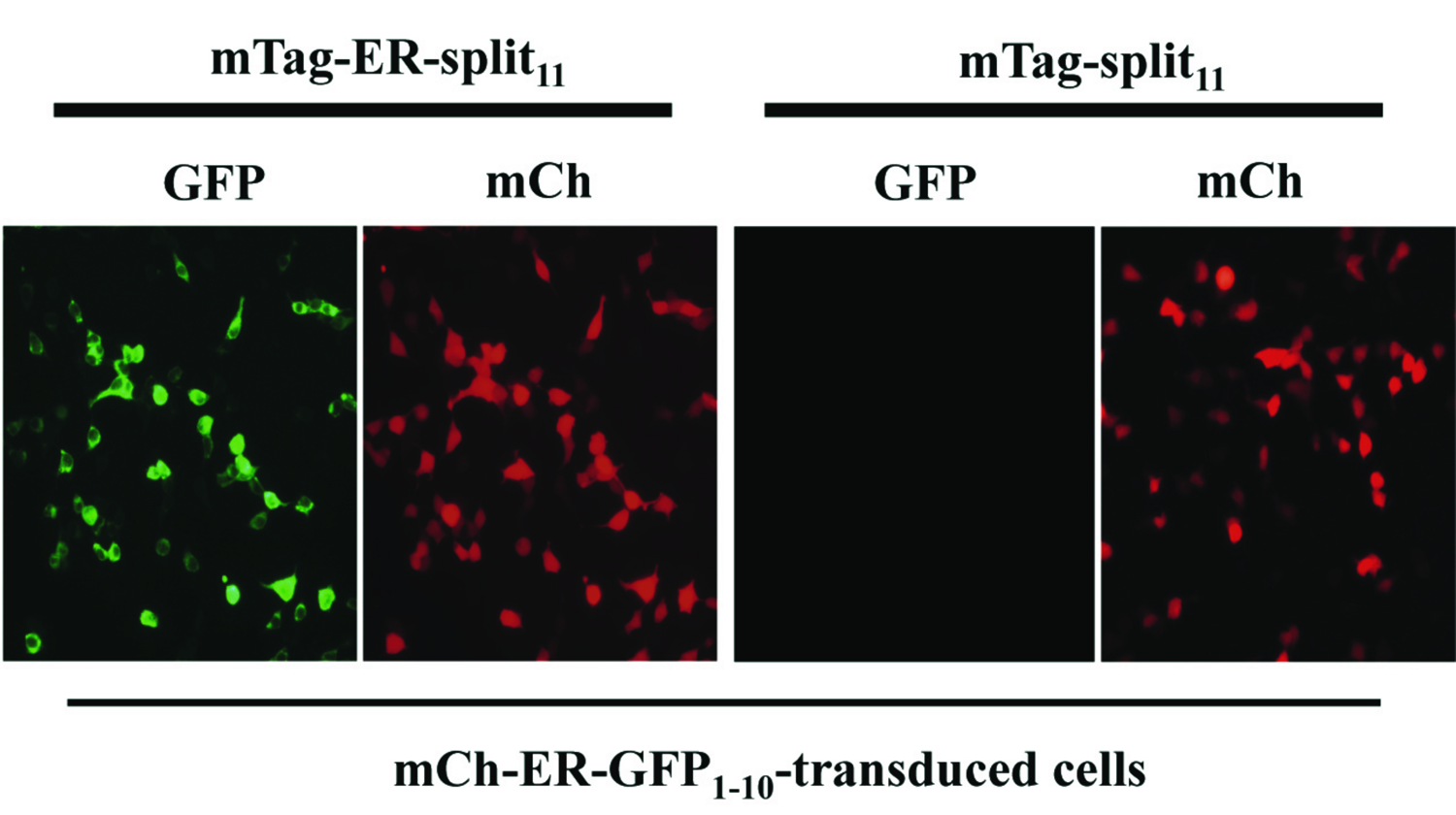

Supplement: Fig. S3 — Specific reconstitution of GFP. [file mbio.01388-23-s0003.tif]
