# Supplementary material for: Establishment of a CPER reverse genetics system for Powassan virus defines attenuating NS1 glycosylation sites and an infectious NS1-GFP11 reporter virus
Source: mBio. 2023 Jul 25;14(4):e01388-23. doi: 10.1128/mbio.01388-23 (PMC10470542; doi:10.1128/mbio.01388-23)
Supplement: Supplemental material — Table S1, Table S2, and legends to Fig. S1 to S3. [file mbio.01388-23-s0004.docx]

**Table S1 CPER POWV Reverse Genetics Viral Rescue**

| Recombinant POWVs | Viral Rescue |
| --- | --- |
| N85Q | **+** |
| N208Q | **+** |
| N224Q | **+** |
| N85Q/N208Q | **-** |
| N85Q/N224Q | **-** |
| N208Q/N224Q | **-** |
| NS1-Split11-C-terminal | **+** |
| NS1-Split11-297 | **-** |

**Table S2 Oligonucleotides**

| Oligos | Sequence |
| --- | --- |
| F1F | CTTGCACGTGTGTGTGGGCACTTTAG |
| F1R | CTCCCAACACTGTGTGAACAGCTTTC |
| F2F | GAAAGCTGTTCACACAGTGTTGGGAG |
| F2R | GTTCCACCTCCGTCAAGTGCAAGTTC |
| F3F | GAACTTGCACTTGACGGAGGTGGAAC |
| F3R | GGGGTCTCATTGAGCAATGTGTAGAC |
| F4F | GTCTACACATTGCTCAATGAGACCCC |
| F4R | CCATTGATAAGTGAAGCCGCTGATCC |
| F5F | GGATCAGCGGCTTCACTTATCAATGG |
| F5R | GCGGGTGTTTTTCCGAGTCACACACC |
| UTR-Linker F | GGTGTGTGACTCGGAAAAACACCCGCGGGTCGGCATGGCATCTCCACCTCC |
| UTR-Linker R | CTAAAGTGCCCACACACACGTGCAAGAAAATCTCGGTTCACTAAACGAGCTCTGCTTATATAGACCTCCC |
| NS1 N85Q F | CGAAGGAGAGGCGCAACTGACCATC |
| NS1 N85Q R | GATGGTCAGTTGCGCCTCTCCTTCG |
| NS1 N208Q F | GAGTTCCTTCCGGCAAGATACTGGCA |
| NS1 N208Q R | TGCCAGTATCTTGCCGGAAGGAACTC |
| NS1 N224Q F | CTGACCTTCGACAATGTACATGGCCA |
| NS1 N224Q R | TGGCCATGTACATTGTCGAAGGTCAG |
| F2A-Split11 R | TACTCATGAAGGACCATGTGGTCACGAGCCATTACCATGGATCGAACCAAGCCACCCTGTGAGTGAACCG |
| F2B-Split11 F | CGTGACCACATGGTCCTTCATGAGTATGTAAATGCTGCCGGTATCACCGACAACGGTGCGATGCTCAGCGAGGG |

**Figure S1.** VeroE6 cells were infected with WT LI9 POWV, recLI9-NS1_N85Q_, recLI9-NS1_N208Q_, and recLI9-NS1_N224Q_ mutant viruses (MOI, 1) and 7 dpi cell RNAs were extracted, and cDNA synthesized using a 3’UTR-reverse primer. Mutants were sequenced and sequencing chromatograms of mutated glycosylation sites are shown versus WT LI9 to validate mutant construction.

**Figure S2.** The POWV NS1 hexamer model was generated by using the cryoEM structure of DENV2 soluble NS1 hexamers (PDB 7WUV) as scaffolding and then relaxed using Rosetta Relax^(101, 102)^. NS1 domains were pseudocolored in blue (β-roll), pink (wing), and yellow (β-ladder). Asparagine N85, N208, and N224 are colored red within the structure.

**Figure S3.** HEK293T cells were PEI co-transfected with cytoplasmic or ER localized GFP11 expression constructs (pQCXIP-mTag-ER-split11 or pQCXIP-mTag-split11) and plasmid pQCXIP-mCh-2A-ER-GFP_1-10_ that expresses ER-translocated GFP_1-10_. Live cell microscopy visualized 2 dpt demonstrates the specific reconstitution of GFP only when GFP11 is ER localized.
